# Ethanol Extract of *Catharanthus roseus* Leaves Exhibits Anticancer Effects on HSC-3 Tongue Cancer Cells Associated with PI3K Suppression and PARP Cleavage

**DOI:** 10.1055/s-0046-1816555

**Published:** 2026-03-14

**Authors:** Ferry Sandra, Melanie Sadono Djamil, Muhammad Ihsan Rizal, Ria Aryani Hayuningtyas, Jackson Dipankara, Nur'amalia Isnaeni, Samuel Ryan Krisdianto, Kerinillia Mochtar, Kyung Hoon Lee

**Affiliations:** 1 Department of Biochemistry and Molecular Biology, Division of Oral Biology, Faculty of Dentistry, Universitas Trisakti, Indonesia; 2 Center of Molecular Biology Study, Faculty of Dentistry, Universitas Trisakti, Jakarta, Indonesia; 3 Department of Oral and Maxillofacial Surgery, Faculty of Dentistry, Universitas Trisakti, Jakarta, Indonesia; 4 Faculty of Dentistry, Universitas Trisakti, Jakarta, Indonesia; 5 Research Institute, Ballys Co. Ltd, Incheon, Republic of Korea

**Keywords:** *Catharanthus roseus*, tongue cancer, HSC-3 cells, apoptosis, PI3K, PARP

## Abstract

**Objective:**

Tongue cancer represents nearly half of all oral cancer cases globally, necessitating the exploration of novel therapeutic agents. *Catharanthus roseus* , known for its well-established anticancer properties, offers a promising alternative. Although *C. roseus* has been studied in several cancer models, its effects in tongue cancer cells, particularly in relation to phosphatidylinositol-3-kinase (PI3K) and poly(adenosine diphosphate-ribose) polymerase (PARP) pathways, have not been evaluated. This study examined the effect of the ethanol extract of *C. roseus* leaf (EECRL) on HSC-3 cells.

**Materials and Methods:**

Cells were treated with various concentrations of EECRL. Cell viability was assessed using the MTT assay, apoptosis was evaluated by sub-G1 analysis, and levels of cleaved-PARP and phosphorylated PI3K were measured using enzyme-linked immunosorbent assay.

**Statistical Analysis:**

Data normality was assessed using the Shapiro–Wilk test. Depending on data distribution, comparisons were performed using one-way analysis of variance followed by Tukey's post hoc test or the Kruskal–Wallis test followed by Dunn's post hoc test. Correction for multiple comparisons was applied using the Holm–Bonferroni method, and adjusted *p* -values were used to determine statistical significance.

**Results:**

EECRL reduced number of viable cells and induced apoptosis in HSC-3 cells in a significant, concentration-dependent manner (adjusted *p* -value < 0.05). Additionally, EECRL significantly decreased the activity of PI3K in a concentration-dependent manner (adjusted *p* -value < 0.05) and significantly increased cleaved-PARP compared with the untreated group (adjusted *p* -value < 0.05).

**Conclusion:**

EECRL reduced viable cell numbers and induced apoptotic features in HSC-3 tongue cancer cells, with effects associated with suppression of PI3K signaling and increased PARP cleavage. These findings suggest that EECRL may have potential as an alternative therapeutic candidate for tongue cancer. However, further mechanistic studies are required to confirm causal pathway involvement.

## Introduction

Tongue cancer, which accounts for nearly 50% of all oral cancer cases, 1 remains a significant global health concern. 2 3 Despite advances in cancer research, the search for alternative therapeutic agents for tongue cancer continues. 4 *Catharanthus roseus* , also referred to as Madagascar periwinkle, has attracted considerable interest owing to its well-documented anticancer properties. 4 5 The alkaloids found in *C. roseus* , particularly vincristine and vinblastine, are renowned for their anticancer effects. 6 These compounds exert their action by inhibiting microtubule formation during cell division, effectively suppressing cancer cell growth. 7 Furthermore, they modulate various signaling pathways to inhibit cancer progression and induce apoptosis. 8

Apoptosis, or programmed cell death, is a critical mechanism for maintaining cellular homeostasis and preventing cancer progression. 9 10 Multiple signaling pathways control this genetically regulated process, one of which is the phosphatidylinositol-3-kinase (PI3K) pathway. 11 The PI3K pathway plays a central role in the regulation of cell proliferation, survival, and apoptosis. 11 12 The PI3K complex comprises three subunits: the regulatory subunits p85 and p55 and the catalytic subunit p110. 13 Dysregulation of the PI3K pathway can lead to uncontrolled cell growth, tumor formation, and apoptosis resistance, making it one of the most frequently disrupted pathways in cancer cells. 14 PI3K also interacts with poly(adenosine diphosphate-ribose) polymerase (PARP), an enzyme involved in DNA repair, cell proliferation, and cell death via poly(ADP-ribosylation). 15 16

Activation of the PI3K pathway inhibits apoptosis by promoting cancer cell survival, primarily through the phosphorylation and inactivation of proapoptotic factors such as Bax and Bad, which stabilize antiapoptotic proteins such as Bcl-2 and Bcl-xL. 17 This prevents cytochrome c release from the mitochondria. 18 When the PI3K pathway is suppressed, Bax and Bad become activated, triggering mitochondrial outer membrane permeabilization and the release of cytochrome c, which initiates the caspase cascade, including caspase-9 and caspase-3, leading to apoptosis. 18 19 PARP, a key player in DNA repair, is cleaved during this process, further committing the cell to apoptosis. 15 16 Akt, a serine/threonine kinase downstream of PI3K, also regulates cell cycle progression by influencing molecules such as p21 and GSK3β. 20 The overexpression or constitutive activation of Akt, which is commonly observed in many cancers, contributes to tumorigenesis and resistance to apoptosis. 21 In addition, Akt modulates key molecules, such as mTOR, which controls cell growth, and FoxO transcription factors, which are involved in apoptosis and cell cycle regulation. 22 23

Although *C. roseus* extracts and derivatives have been studied in various cancer models, including nanoparticle formulations, the mechanisms underlying their effects in tongue cancer remain unclear. In particular, the role of *C. roseus* extracts in modulating PI3K and PARP activation in human squamous cell carcinoma (HSC)-3 tongue cancer cells has not yet been investigated. Therefore, this study aimed to investigate the effects of the ethanolic extract of *C. roseus* leaves (EECRL) on HSC-3 cells, focusing on its potential to reduce viable cell number and promote apoptosis as well as the PI3K and PARP activation.

## Materials and Methods

### Ethanol Extract of *Catharanthus roseus* Leaves Preparation

*Catharanthus roseus* leaves were obtained and authenticated at the Indonesian Agency for Agricultural Instrument Standardization of Spices, Medicinal, and Aromatic Crops, Ministry of Agriculture, Republic of Indonesia. Plant identification and taxonomic determination were conducted at the same institution.

A total of 350 g of dried, finely chopped leaves (simplicia) was macerated in 5 L of 70% ethanol for 24 hours at room temperature. The extract was filtered, and ethanol was removed using a rotary evaporator to obtain 126 g of crude extract (yield ≈ 36% w/w). The crude extract was stored at 4°C under standard laboratory conditions.

For in vitro experiments, 250 mg of crude extract was dissolved in 150 mL of complete culture medium and further diluted to obtain the desired working concentrations. All experiments were conducted using a single extract batch to minimize batch-to-batch variation and ensure reproducibility.

### Phytochemical Composition Analysis of Ethanol Extract of *Catharanthus roseus* Leaves

The phytochemical composition of EECRL was analyzed using an Agilent 1200 HPLC system (Agilent Technologies, Santa Clara, California, United States) coupled with 3200 QTRAP LC-MS/MS (liquid chromatography tandem mass spectrometry; AB Sciex, Framingham, Massachusetts, United States), following established protocol. 7 The analysis focused on indole alkaloids, which are the primary bioactive constituents of *C. roseus* . The LC-MS/MS data were analyzed in a semiquantitative manner, and the relative abundance of each compound was expressed as a percentage of the total alkaloid content in the extract.

### HSC-3 Cell Culture

HSC-3 cells were cultured as previously described, 18 with minor modifications. The HSC-3 cell line was grown in Dulbecco's Modified Eagle Medium (DMEM) containing 50 μg/mL streptomycin, 50 U/mL penicillin, and 10% fetal bovine serum. Cultures were maintained in a humidified incubator at 37°C with 5% CO _2_ . Upon reaching approximately 80% confluence, cells were detached using trypsin-EDTA solution. The number of cells was determined using a Countess 3 Automated Cell Counter (Thermo Fisher Scientific, Waltham, Massachusetts, United States) prior to seeding to ensure equal cell density across wells.

### Viable Cell Number Assay

Viable cell assessment was conducted using a sequential approach combining a metabolic activity–based assay and reference cell counting. The 3-(4,5-dimethylthiazol-2-yl)-2,5-diphenyltetrazolium bromide (MTT) assay (Sigma-Aldrich, St. Louis, Missouri, United States) was performed following a previously established method. 18 To ensure data reliability and reproducibility, the MTT assay was performed multiple times, and the data presented in this study were generated from six independent experimental replicates ( *n* = 6).

HSC-3 cells (5 × 10 ^3^ cells/well) were seeded in 96-well plates and subjected to 12 hours of nutrient deprivation, followed by treatment with or without 1.5625, 3.125, 6.25, 12.5, 25, or 50 μg/mL of EECRL, or 3 μM doxorubicin (Sigma-Aldrich) for 24 hours. Untreated cells cultured in DMEM alone served as the negative control.

Subsequently, 100 μL of MTT solution (0.5 mg/mL) was added to each well and incubated for 4 hours at 37°C. The medium was removed, and the resulting formazan crystals were dissolved in 100 μL of dimethyl sulfoxide (Sigma-Aldrich). Absorbance was measured at 570 nm using a microplate reader (Bio-Rad, Hercules, California, United States). The MTT assay was used to assess relative cellular metabolic activity, which was considered proportional to the viable cell proportion within the tested concentration range.

To relate MTT absorbance values to viable cell proportion, an additional untreated reference group was concurrently analyzed using the Countess 3 Automated Cell Counter (Thermo Fisher Scientific, Waltham, Massachusetts, United States) based on trypan blue exclusion. The viable cell number obtained from this untreated group was used solely as a reference for converting MTT absorbance values into relative percentages of viable cells, assuming a linear relationship between metabolic activity and cell number under these experimental conditions. Thus, MTT results are reported as relative viable cell percentages rather than absolute cell numbers.

### Sub-G1 Assay

The Sub-G1 assay was used to evaluate the cytotoxic effects of EECRL-induced apoptosis, based on standard procedure. 18 HSC-3 cells (1.5 × 10 ^3^ cells/well) were seeded in 48-well plates, deprived of nutrients for 12 hours, and treated with or without 6.25, 12.5, 25, or 50 μg/mL of EECRL, or 3 μM doxorubicin for 24 hours. Doxorubicin was used as a positive control for apoptosis. Cells were collected and incubated in 450 μL of a hypotonic fluorochrome solution containing 50 μg/mL propidium iodide (Sigma-Aldrich), 0.1% sodium citrate (Sigma-Aldrich), and 0.1% Triton X-100 (Sigma-Aldrich). After 30 minutes of incubation in the dark, fluorescence was analyzed using a FACSCanto II flow cytometer (Becton Dickinson, Franklin Lakes, New Jersey, United States), measured at fluorescence length (FL)-2, 400 events/s, for 10,000 total events. After assay optimization, Sub-G1 analysis was conducted with six independent biological replicates for final data analysis. The Sub-G1 fraction was interpreted as an indicator of apoptosis-associated changes rather than a definitive measure of apoptosis.

### Enzyme-Linked Immunosorbent Assay Analysis of PI3K

Levels of phosphorylated and total PI3K p85 were measured using a cell-based enzyme-linked immunosorbent assay (ELISA kit; Abcam, Cambridge, UK). Cells were treated with EECRL for 1 hour prior to fixation with 4% formaldehyde and permeabilization with 1% Triton X-100. Absorbance was measured at 450 nm. Wortmannin was used as a PI3K inhibitor control. This analysis was performed with three independent biological replicates. Results were expressed as the p-PI3K/PI3K absorbance ratio to reflect relative changes in PI3K activation.

### Enzyme-Linked Immunosorbent Assay Analysis of PARP

Cleaved-PARP [214/215] levels were quantified using a sandwich ELISA kit (Invitrogen, Waltham, Massachusetts, United States). The cells were treated for 6 hours prior to lysis. Briefly, the treated cells were lysed using cell extraction buffer (Invitrogen), supplemented with phenylmethylsulfonyl fluoride and a protease inhibitor cocktail according to the manufacturer's instructions. The resulting lysates were centrifuged, and the total protein concentration was quantified using the Bradford protein assay to normalize the protein input across samples (Bio-Rad). Equal amounts of protein were loaded into the wells and incubated with a cleaved-PARP-specific antibody. After incubation, the wells were washed, and an anti-rabbit IgG HRP solution was added. After another incubation and washing, the TMB substrate was added. The reaction was stopped, and the change in color was measured at 450 nm. The assay specifically detected the human cleaved-PARP [214/215] fragment with a sensitivity of <0.062 ng/mL. Doxorubicin was used as a positive control for PARP cleavage, whereas Z-DEVD-FMK (R&D Systems, Minneapolis, Minnesota, United States), a caspase-3 inhibitor upstream of PARP, served as an inhibitory control. ELISA experiments were performed with three independent biological replicates.

### Statistical Analysis

Data normality was assessed using the Shapiro–Wilk test. For normally distributed data, one-way analysis of variance followed by Tukey's post hoc test was applied, whereas non-normally distributed data were analyzed using the Kruskal–Wallis test followed by Dunn's post hoc test for pairwise comparisons. Initial statistical analyses were performed using SPSS version 23.0. Correction for multiple comparisons was applied using the Holm–Bonferroni method, and adjusted *p* -values are reported. An adjusted *p* -value < 0.05 was considered statistically significant. IC _50_ values were calculated by nonlinear regression using an exponential curve-fitting model in Microsoft Excel.

## Results

### Phytochemical Composition of Ethanol Extract of *Catharanthus roseus* Leaves

EECRL analysis confirmed the presence of several indole alkaloids, which are the principal bioactive constituents of *C. roseus* . The major compounds identified were vinblastine, vincristine, catharanthine, vindoline, ajmalicine, and serpentine. Among these, vindoline (24.3%) and catharanthine (18.2%) were the most abundant alkaloids, as summarized in Table 1 . These compounds are widely recognized for their antiproliferative and proapoptotic activities, supporting the biological relevance of EECRL in anticancer studies.

**Table 1:** Major alkaloids identified in the ethanolic extract of *Catharanthus roseu* *s* leaves

Compound	Relative abundance (%)
Vinblastine	4.1
Vincristine	2.6
Catharanthine	18.2
Vindoline	24.3
Ajmalicine	14.7
Serpentine	11.7
Others (minor alkaloids)	24.4

### Ethanol Extract of *Catharanthus roseus* Leaves Reduced the Number of Viable HSC-3 Cells

**Fig. 1:**
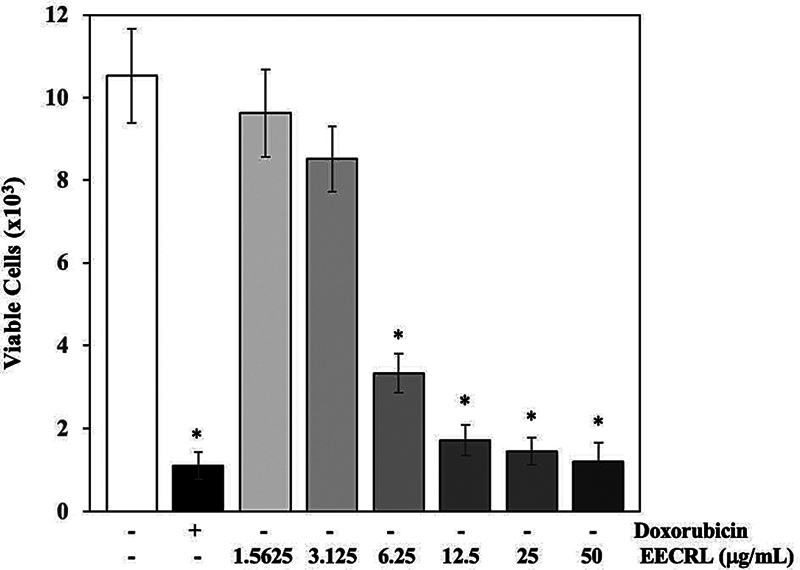
EECRL reduced the number of viable HSC-3 cells in a concentration-dependent manner. After a 12-hour starvation period, HSC-3 cells were treated with or without 3 μM doxorubicin or various EECRL concentrations for 24 hours. The number of viable cells were measured using the MTT assay, as described in the Materials and Methods section. Data are presented as mean ± standard deviation ( *n* = 6). *Significant (adjusted *p* -value < 0.05) compared with the untreated group. EECRL, ethanolic extract of Catharanthus *roseus* leaves.

The number of viable HSC-3 cells in the doxorubicin-treated group (1,100 ± 331) was significantly lower (adjusted *p* -value < 0.05) than that in the untreated group (10,531 ± 1,141) ( Fig. 1 and Table 2 ). EECRL treatment significantly reduced the number of viable HSC-3 cells in a concentration-dependent manner (adjusted *p* -value < 0.05), with higher concentrations leading to lower number of viable cells. In the 1.5625, 3.125, 6.25, 12.5, 25, and 50 μg/mL EECRL-treated groups, the numbers of viable cells were 9,626 ± 1,061; 8,513 ± 787; 3,323 ± 466; 1,727 ± 375; 1,454 ± 336; and 1,200 ± 452, respectively. The values of 6.25, 12.5, 25, and 50 μg/mL EECRL-treated groups were significantly lower than those in the untreated group (adjusted *p* -value < 0.05). Based on the data, the IC _50_ was calculated at 5.65 μg/mL, corresponding to the concentration of EECRL that reduced viable cell numbers to approximately half of the untreated group.

**Table 2:** Ethanolic extract of *Catharanthus roseu* *s* leaves reduced the number of viable HSC-3 cells

Group	Viable cells	Adj. *p* -value	
Untreated	10,531 ± 1,141	vs. Doxorubicin	0.028 a
		vs. 1.5625 µg/mL EECRL	1
		vs. 3.125 µg/mL EECRL	1
		vs. 6.25 µg/mL EECRL	0.028 a
		vs. 12.5 µg/mL EECRL	0.028 a
		vs. 25 µg/mL EECRL	0.028 a
		vs. 50 µg/mL EECRL	0.028 a
Doxorubicin	1,100 ± 331	vs. 1.5625 µg/mL EECRL	0.028 a
		vs. 3.125 µg/mL EECRL	0.028 a
		vs. 6.25 µg/mL EECRL	0.05
		vs. 12.5 µg/mL EECRL	1
		vs. 25 µg/mL EECRL	1
		vs. 50 µg/mL EECRL	1
1.5625 µg/mL EECRL	9,626 ± 1,061	vs. 3.125 µg/mL EECRL	1
		vs. 6.25 µg/mL EECRL	0.028 a
		vs. 12.5 µg/mL EECRL	0.028 a
		vs. 25 µg/mL EECRL	0.028 a
		vs. 50 µg/mL EECRL	0.028 a
3.125 µg/mL EECRL	8,513 ± 787	vs. 6.25 µg/mL EECRL	0.028 a
		vs. 12.5 µg/mL EECRL	0.028 a
		vs. 25 µg/mL EECRL	0.028 a
		vs. 50 µg/mL EECRL	0.028 a
6.25 µg/mL EECRL	3,323 ± 466	vs. 12.5 µg/mL EECRL	0.028 a
		vs. 25 µg/mL EECRL	0.028 a
		vs. 50 µg/mL EECRL	0.028 a
12.5 µg/mL EECRL	1,727 ± 375	vs. 25 µg/mL EECRL	1
		vs. 50 µg/mL EECRL	1
25 µg/mL EECRL	1,454 ± 336	vs. 50 µg/mL EECRL	1
50 µg/mL EECRL	1,200 ± 452		

Abbreviation: EECRL, ethanolic extract of *Catharanthus roseus* leaves.

a statistically significant difference based on adjusted *p* -values (p_adj < 0.05).

### Ethanol Extract of *Catharanthus roseus* Leaves Increased the Percentage of Apoptotic HSC-3 Cells

**Fig. 2:**
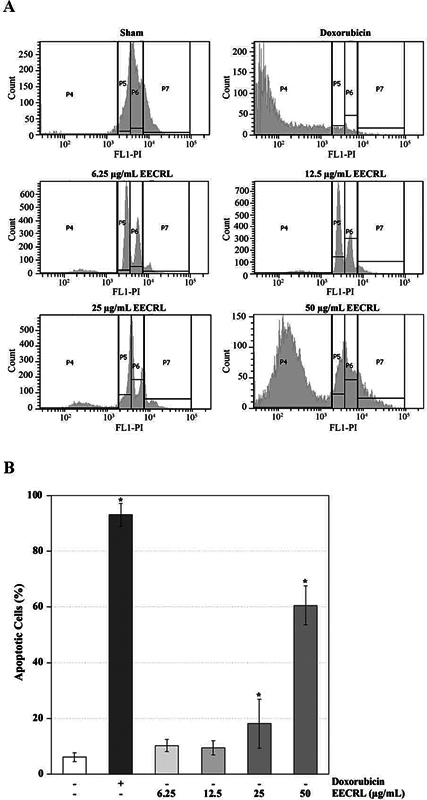
EECRL increased apoptotic HSC-3 cell percentage in a concentration-dependent manner. After a 12-hour starvation period, HSC-3 cells were treated with or without 3 μM doxorubicin or various EECRL concentrations for 24 hours. The percentage of apoptotic cells was measured using the Sub-G1 assay, as described in the Materials and Methods section. ( **A** ) Histograms showing the distribution of treated HSC-3 cells across Sub-G1, G1, S, and G2/M phases. ( **B** ) The percentage of apoptotic HSC-3 cells. Data are presented as mean ± standard deviation ( *n* = 6). *Significant (adjusted *p* -value < 0.05) compared with the untreated group. EECRL, ethanolic extract of *Catharanthus roseus* leaves; P4: Sub-G1 phase; P5: G1 phase; P6: S phase; P7: G2/M phase.

Cell cycle analysis revealed an increase in the Sub-G1 population, indicative of apoptotic cell death. The histogram ( Fig. 2A and Table 3 ) showed a markedly higher Sub-G1 fraction in the doxorubicin-treated group compared with the untreated group. At 6.25 µg/mL EECRL, only a slight increase in Sub-G1 cells was observed, whereas progressive increases were evident at higher concentrations (12.5, 25, and 50 µg/mL). The quantitative percentage of apoptotic cells is presented in Fig. 2B . The percentage of apoptotic HSC-3 cells in the doxorubicin-treated group (93.08 ± 4.09%) was significantly higher than that in the untreated group (6.08 ± 1.59%) (adjusted *p* -value < 0.05). EECRL treatment significantly increased apoptotic cell percentages in a concentration-dependent manner (adjusted *p* -value < 0.05). In the 6.25, 12.5, 25, and 50 µg/mL EECRL-treated groups, apoptotic cell percentages were 10.27 ± 2.16%, 9.40 ± 2.53%, 18.10 ± 8.79%, and 60.55 ± 7.00%, respectively. While the increases at 6.25 and 12.5 µg/mL were not statistically significant compared with the untreated group, significant apoptosis induction was observed at 25 and 50 µg/mL (adjusted *p* -value < 0.05).

**Table 3:** Ethanolic extract of *Catharanthus roseu* *s* leaves increased the percentage of apoptotic HSC-3 cells

Group	Apoptotic cells (%)	Adj. *p* -value	
Untreated	6.08 ± 1.59	vs. Doxorubicin	0.014 ^a^
		vs. 6.25 µg/mL EECRL	1
		vs. 12.5 µg/mL EECRL	1
		vs. 25 µg/mL EECRL	0.024 ^a^
		vs. 50 µg/mL EECRL	0.0015 ^a^
Doxorubicin	93.08 ± 4.09	vs. 6.25 µg/mL EECRL	0.014 ^a^
		vs. 12.5 µg/mL EECRL	0.014 ^a^
		vs. 25 µg/mL EECRL	0.014 ^a^
		vs. 50 µg/mL EECRL	0.014 ^a^
6.25 µg/mL EECRL	10.27 ± 2.16	vs. 12.5 µg/mL EECRL	1
		vs. 25 µg/mL EECRL	0.708
		vs. 50 µg/mL EECRL	0.014 ^a^
12.5 µg/mL EECRL	9.40 ± 2.53	vs. 25 µg/mL EECRL	0.49
		vs. 50 µg/mL EECRL	0.014 ^a^
25 µg/mL EECRL	18.10 ± 8.79	vs. 50 µg/mL EECRL	0.014 ^a^
50 µg/mL EECRL	60.55 ± 7.00		

Abbreviation: EECRL, ethanolic extract of *Catharanthus roseus* leaves.

### Ethanol Extract of *Catharanthus roseus* Leaves Reduced PI3K Activity in HSC-3 Cells

**Fig. 3:**
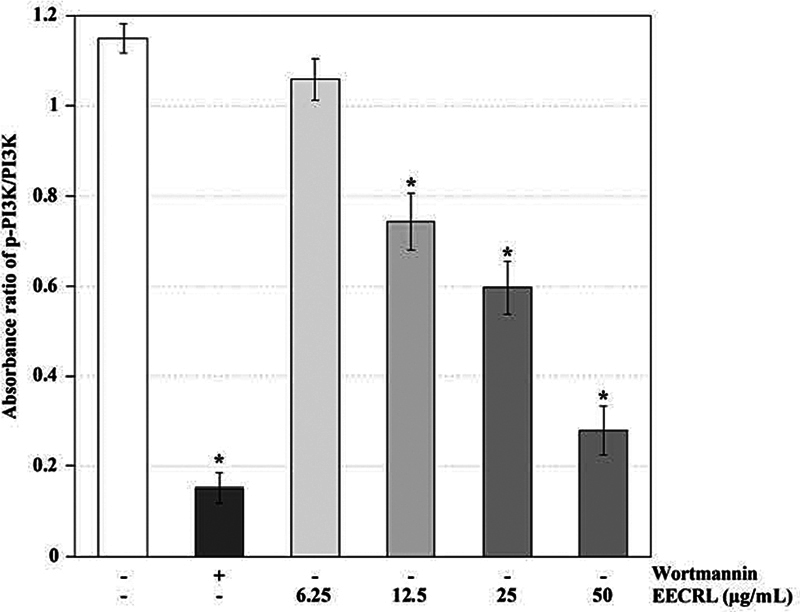
EECRL reduced the p-PI3K/PI3K absorbance ratio in HSC-3 cells in a concentration-dependent manner. Cells were seeded at a density of 5,000 cells/well in a 96-well plate and cultured for 2 days. After that, the cells were starved for 12 hours, then treated with or without 1 µM wortmannin or various concentrations of EECRL for 1 hour. The absorbances of p-PI3K and PI3K were measured using the ELISA analysis of PI3K, as described in the Materials and Methods section. Data are presented as mean ± standard deviation ( *n* = 3). *Significant (adjusted *p* -value < 0.05) compared with the untreated group. EECRL, ethanolic extract of *Catharanthus roseus* leaves; ELISA, enzyme-linked immunosorbent assay.

The p-PI3K/PI3K absorbance ratio in the wortmannin-treated group (0.152 ± 0.033) was significantly lower (adjusted *p* -value < 0.05) than that in the untreated group (1.149 ± 0.033) ( Fig. 3 and Table 4 ). EECRL treatment significantly reduced the p-PI3K/PI3K absorbance ratio in a concentration-dependent manner (adjusted *p* -value < 0.05). The absorbance ratios of p-PI3K/PI3K for the 6.25, 12.5, 25, and 50 μg/mL EECRL-treated groups were 1.059 ± 0.046, 0.745 ± 0.063, 0.599 ± 0.059, and 0.280 ± 0.054, respectively. The absorbance ratios of p-PI3K/PI3K for the 12.5, 25, and 50 μg/mL EECRL-treated groups were significantly lower than those in the untreated group (adjusted *p* -value < 0.05).

**Table 4:** Ethanolic extract of *Catharanthus roseu* *s* leaves reduced the p-PI3K/PI3K absorbance ratio

Group	Absorbance ratio of p-PI3K to PI3K	Adj. *p* -value	
Untreated	1.149 ± 0.033	vs. Wortmannin	0.015 ^a^
		vs. 6.25 µg/mL EECRL	0.293
		vs. 12.5 µg/mL EECRL	0.015 ^a^
		vs. 25 µg/mL EECRL	0.015 ^a^
		vs. 50 µg/mL EECRL	0.015 ^a^
Wortmannin	0.152 ± 0.033	vs. 6.25 µg/mL EECRL	0.015 ^a^
		vs. 12.5 µg/mL EECRL	0.015 ^a^
		vs. 25 µg/mL EECRL	0.015 ^a^
		vs. 50 µg/mL EECRL	0.146
6.25 µg/mL EECRL	1.059 ± 0.046	vs. 12.5 µg/mL EECRL	0.015 ^a^
		vs. 25 µg/mL EECRL	0.015 ^a^
		vs. 50 µg/mL EECRL	0.015 ^a^
12.5 µg/mL EECRL	0.745 ± 0.063	vs. 25 µg/mL EECRL	0.099
		vs. 50 µg/mL EECRL	0.015 ^a^
25 µg/mL EECRL	0.599 ± 0.059	vs. 50 µg/mL EECRL	0.015 ^a^
50 µg/mL EECRL	0.280 ± 0.054		

Abbreviation: EECRL, ethanolic extract of *Catharanthus roseus* leaves.

### Ethanol Extract of *Catharanthus roseus* Leaves Reduced Cleaved-PARP Levels in HSC-3 Cells

**Fig. 4:**
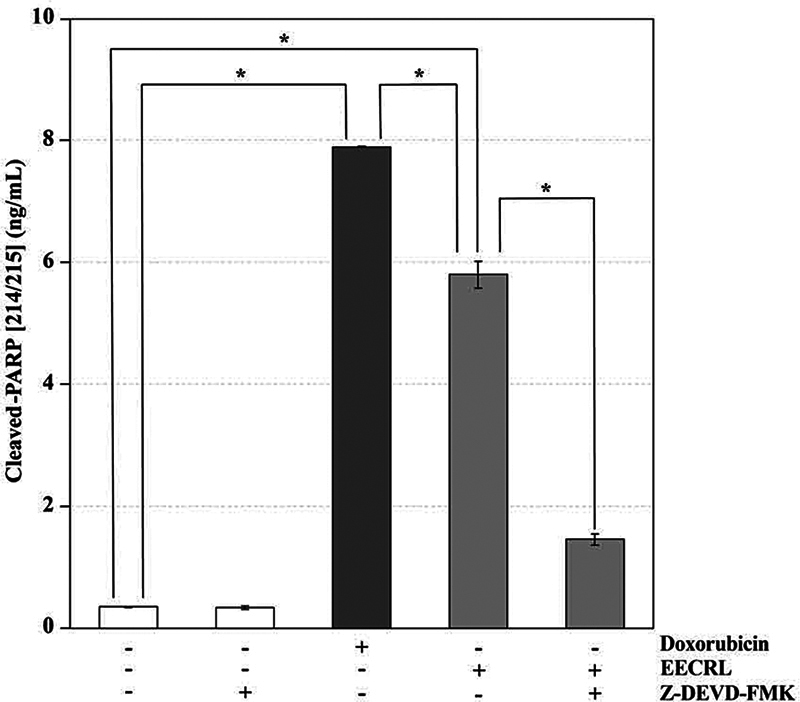
EECRL reduced the cleaved-PARP level. Cells were seeded at a density of 5,000 cells/well in a 96-well plate and cultured for 2 days. After that, the cells were starved for 12 hours, then treated with or without 3 μM doxorubicin, 50 μg/mL EECRL or 100 μM Z-DEVD-FMK for 6 hours. The absorbance of cleaved-PARP was measured using the ELISA analysis of PARP, as described in the Materials and Methods section. Data are presented as mean ± standard deviation ( *n* = 3). *Significant (adjusted *p* -value < 0.05). EECRL, ethanolic extract of *Catharanthus roseus* leaves; ELISA, enzyme-linked immunosorbent assay.

The cleaved-PARP levels in the untreated group (0.345 ± 0.009 ng/mL) and the group treated with Z-DEVD-FMK alone (0.325 ± 0.032 ng/mL) were low ( Fig. 4 and Table 5 ). Meanwhile, the doxorubicin-treated group showed a significant (adjusted *p* -value < 0.05) in cleaved-PARP levels (7.893 ± 0.011 ng/mL) compared with the untreated group. Treatment with 50 μg/mL EECRL also significantly (adjusted *p* -value < 0.05) increased cleaved-PARP levels (5.802 ± 0.219 ng/mL) compared with the untreated group, confirming the induction of apoptosis. Additionally, cotreatment of 50 μg/mL EECRL with Z-DEVD-FMK further reduced the cleaved-PARP levels (1.451 ± 0.094 ng/mL), which were significantly (adjusted *p* -value < 0.05) lower than those in the 50 μg/mL EECRL-treated group.

**Table 5:** Ethanolic extract of *Catharanthus roseu* *s* leaves reduced the cleaved-PARP level

Group	The level of cleaved-PARP (ng/mL)	Adj. *p* -value	
Untreated	0.345 ± 0.009	vs. Untreated + Z-DEVD-FMK	0.999
		vs. Doxorubicin	0.01 ^a^
		vs. 50 µg/mL EECRL	0.01 ^a^
		vs. 50 µg/mL EECRL + Z-DEVD-FMK	0.01 ^a^
Z-DEVD-FMK	0.325 ± 0.032	vs. Doxorubicin	0.01 ^a^
		vs. 50 µg/mL EECRL	0.01 ^a^
		vs. 50 µg/mL EECRL + Z-DEVD-FMK	0.01 ^a^
Doxorubicin	7.893 ± 0.011	vs. 50 µg/mL EECRL	0.01 ^a^
		vs. 50 µg/mL EECRL + Z-DEVD-FMK	0.01 ^a^
50 µg/mL EECRL	5.802 ± 0.219	vs. 50 µg/mL EECRL + Z-DEVD-FMK	0.01 ^a^
50 µg/mL EECRL + Z-DEVD-FMK	1.451 ± 0.094		

Abbreviation: EECRL, ethanolic extract of *Catharanthus roseus* leaves.

## Discussion

EECRL demonstrated a dual effect on HSC-3 tongue cancer cells by reducing viable cell number and increasing the Sub-G1 population, consistent with apoptosis-associated cell death. EECRL demonstrated dual effects on HSC-3 tongue cancer cells, reducing viable cell numbers and exhibiting apoptosis-related cellular changes, thereby impairing cancer cell proliferation. This dual action suggests biological activity of the extract, particularly at higher concentrations. The MTT assay revealed an EECRL-concentration-dependent reduction in viable HSC-3 cell number, consistent with a previous report on the anticancer properties of *C. roseus* extracts. Alkaloids such as vincristine and vinblastine, disrupt microtubule formation during mitosis, and inhibit cell proliferation. 23 24 In a similar manner, EECRL suppressed HSC-3 cell growth. Based on the data, the IC _50_ value was calculated as 5.65 µg/mL, indicating strong cytotoxicity activity. 25 26 The findings align with the cytotoxic effects of *C. roseus* derivatives reported in other studies. *Catharanthus roseus* -derived gold nanoparticles in cervical cancer (HeLa) cells significantly reduced cell proliferation at concentrations ranging from 1 to 15 µg/mL, with an IC _50_ value of 5 µg/mL. 27 Collectively, these results support the anticancer-associated activity of *C. roseus* extracts and its derivatives against tongue cancer cells.

Sub-G1 phase analysis revealed a significant increase in the Sub-G1 cell population following EECRL treatment, particularly at higher concentrations, indicating apoptosis-associated DNA fragmentation rather than definitive apoptosis. This observation is consistent with previous studies on T47D breast cancer cells, where *C. roseus* extracts increased apoptosis-related markers. At concentration of 6.25 µg/mL, 31.26% of T47D cells were reported in the Sub-G1 fraction. 28 These findings further support the apoptosis-associated effects of *C. roseus* extract and are consistent with the present observations in HSC-3 tongue cancer cell.

Furthermore, the ELISA results indicated that EECRL treatment reduced PI3K activity in HSC-3 cells, as evidenced by a decrease in the p-PI3K/PI3K absorbance ratio. The PI3K pathway is frequently dysregulated in cancer and is strongly associated with apoptosis resistance, enhanced survival signaling, and uncontrolled cell proliferation. 29 30 The observed reduction in PI3K phosphorylation suggests that EECRL may modulate PI3K-related signaling pathways, which are commonly implicated in cancer cell survival. The concentration-dependent decrease in the p-PI3K/PI3K ratio indicated that EECRL-induced cytotoxicity is associated with alterations in this pathway, rather than confirming a direct causal mechanism. In line with these observations, a network pharmacology-based study identified the PI3K pathway as a potential molecular target of *C. roseus* compounds. 31 Downstream of PI3K, activation of Akt and its associated signaling network has been widely implicated in cancer cell survival, proliferation, and resistance to apoptosis. Although downstream effectors such as Akt, mTOR, or inhibitor of apoptosis proteins were not directly assessed in the present study, suppression of PI3K signaling is commonly linked to attenuation of these survival pathways. Therefore, the observed reduction in PI3K phosphorylation in EECRL-treated cells is compatible with a broader suppression of PI3K-associated survival signaling, rather than definitive proof of a single linear pathway.

PARP cleavage is a widely recognized as a hallmark of apoptosis. 32 33 In the present study, cleaved-PARP levels were elevated in the EECRL-treated group compared with the untreated group, indicating increased apoptosis-associated activity. At 50 μg/mL, EECRL produced a noticeable proapoptotic-associated response, although cleaved-PARP levels remained lower than those induced by doxorubicin, suggesting moderate efficacy. Importantly, cotreatment with the caspase-3 inhibitor Z-DEVD-FMK significantly reduced cleaved-PARP levels, supporting the involvement of caspase-dependent mechanisms in EECRL-associated apoptotic signaling. Previous studies have shown that *C. roseus* extracts can influence apoptotic signaling by upregulating proapoptotic factors such as Bax 34 35 and downregulating antiapoptotic proteins such as Bcl-2, 27 which is consistent with the apoptosis-associated effects observed in this study.

Despite these findings, the precise molecular mechanisms underlying EECRL-induced cytotoxicity and apoptosis-associated changes remain to be fully elucidated. While reduced PI3K activation and increased PARP cleavage are compatible with apoptosis-related processes, additional experiments such as Annexin V/PI staining, caspase-3 or caspase-7 activity assays, or pathway-specific inhibition studies would be required to confirm direct mechanistic involvement. Furthermore, as this study was conducted using a single tongue cancer cell line (HSC-3) and did not include normal oral cell comparators, the conclusions are necessarily limited to in vitro observations, and further studies are needed to assess selectivity and potential therapeutic relevance.

## Conclusion

EECRL could have a potential anticancer effect against HSC-3 tongue cancer cells by reducing viable cell number and inducing apoptosis in a concentration-dependent manner. These effects were associated with decreased PI3K activity and increased PARP cleavage, suggesting the involvement of apoptosis-related pathways. However, further mechanistic and comparative studies are required to confirm direct pathway involvement and to evaluate selectivity and therapeutic relevance.
